# Spherical Convolutional Neural Networks for Survival Rate Prediction in Cancer Patients

**DOI:** 10.3389/fonc.2022.870457

**Published:** 2022-04-27

**Authors:** Fabian Sinzinger, Mehdi Astaraki, Örjan Smedby, Rodrigo Moreno

**Affiliations:** ^1^ Division of Biomedical Imaging, Department of Biomedical Engineering and Health Systems, KTH Royal Institute of Technology, Stockholm, Sweden; ^2^ Karolinska Institutet, Department of Oncology-Pathology, Karolinska Universitetssjukhuset, Stockholm, Sweden

**Keywords:** lung cancer, tumor segmentation, spherical convolutional neural network, survival rate prediction, deep learning, Cox Proportional Hazards, DeepSurv

## Abstract

**Objective:**

Survival Rate Prediction (SRP) is a valuable tool to assist in the clinical diagnosis and treatment planning of lung cancer patients. In recent years, deep learning (DL) based methods have shown great potential in medical image processing in general and SRP in particular. This study proposes a fully-automated method for SRP from computed tomography (CT) images, which combines an automatic segmentation of the tumor and a DL-based method for extracting rotational-invariant features.

**Methods:**

In the first stage, the tumor is segmented from the CT image of the lungs. Here, we use a deep-learning-based method that entails a variational autoencoder to provide more information to a U-Net segmentation model. Next, the 3D volumetric image of the tumor is projected onto 2D spherical maps. These spherical maps serve as inputs for a spherical convolutional neural network that approximates the log risk for a generalized Cox proportional hazard model.

**Results:**

The proposed method is compared with 17 baseline methods that combine different feature sets and prediction models using three publicly-available datasets: Lung1 (n=422), Lung3 (n=89), and H&N1 (n=136). We observed comparable C-index scores compared to the best-performing baseline methods in a 5-fold cross-validation on Lung1 (0.59 ± 0.03 vs. 0.62 ± 0.04). In comparison, it slightly outperforms all methods in inter-data set evaluation (0.64 vs. 0.63). The best-performing method from the first experiment reduced its performance to 0.61 and 0.62 for Lung3 and H&N1, respectively.

**Discussion:**

The experiments suggest that the performance of spherical features is comparable with previous approaches, but they generalize better when applied to unseen datasets. That might imply that orientation-independent shape features are relevant for SRP. The performance of the proposed method was very similar, using manual and automatic segmentation methods. This makes the proposed model useful in cases where expert annotations are not available or difficult to obtain.

## Introduction

The objective of *Survival Rate Prediction* (SRP) is to estimate the time until a well-defined “terminal event”, which occurs in some, but not necessarily all, cases. For cancer patients, the terminal event may be the death of the patient (“overall survival”), relapse, or progression of the disease (“relapse-free survival” or “progression-free survival”, respectively). It has been shown that image-based characteristics of tumors such as shape, size and texture are associated with malignancy (1). A research avenue that has been explored in the last few years is whether those image-based tumor characteristics can also be used for predicting the survival of cancer patients (2). Survival rate prediction (SRP) from the shape, size, and texture of the tumor is challenging. First, it is not clear if imaging information alone is enough for SRP. Moreover, the prediction might be affected by different factors, including image acquisition parameters, inaccurate segmentation masks, the selected features used for the prediction, the prediction model itself, as well as the presence of right-censored data. Although clinical trials are often relied on clinical assessments like molecular profiling to conduct the survival analysis (3), such information is not always accessible.

While SRP could be framed as a regression-type problem, that is, to predict the time from the last observation to the terminal event, a practical difficulty is that part of the longitudinal data of patients is missing in training datasets for SRP in cancer. More specifically, these datasets usually contain right-censored data, which means that the start of the observation period is known for all data points, but the definitive end of the observation point might be missing for some cases. Consider a dataset where some patients were still alive when the study ended. In such an example, there would be a lower boundary of the survival times, namely, the last known date of record, which is lower than the definitive time of death for some cases. Other reasons for right-censorship in practice could be that patients dropped out of the study and did not have a time of death reported. However, it should be noted that exclusion of such cases is not recommended since that might bias the analysis towards the more lethal cases.

In the past, SRP was usually performed on small feature sets of descriptive statistics or clinical assessments (4). used ensemble data mining to train an outcome calculator on clinical data including features such as patient age at diagnosis, cancer grade, lymph node involvement, among many others. When working with imaging data, *radiomics* (5) provides a catalog of standard methods to extract such statistics automatically. These radiomics features can be used in CoxPH models (4) and other prediction methods such as decision trees, rule-based classification, or naive Bayes (6). More recently, *deep learning* (DL)-based methods have outperformed conventional algorithms in the field of image processing in general (7) and in image-based SRP in particular (8, 9). Among the first approaches using DL, Faraggi and Simon (10) proposed a feed-forward neural network for a non-linear risk-score approximation. A more recent example is DeepSurv, which provides a general framework for DL-based SRP (11).

Since the introduction of DL-based SRP, a vast body of work has been published where different DL algorithms have been applied to diverse modalities and features from various organs. Some examples include SPR for gastric cancer (12), cervical cancer (13), colorectal cancer (14), liver cancer (15), breast cancer (16) and oral cancer (17). In particular, this study is focused on non-small cell lung cancer (NSCLC) SRP. Previous studies from recent years have already shown the potential of DL models for survival analysis of lung cancer patients (18–21).

In some studies [e.g (22). and (23)], features from different modalities including imaging, radiomic features, clinical data, and molecular information, were combined as inputs to improve the performance of DL-based SRP models. While such multimodal prediction pipelines are theoretically superior to single modality-based predictions, the requirement for the respective data availability can be a disadvantage for the application in clinical practice. The financial cost of additional laboratory testing and expert clinical staging and tumor segmentation is another limiting factor of multimodal techniques. In addition, those approaches can only be applied to sites where the required data can be collected. Thus, it is clinically relevant to develop an SRP pipeline that requires only the CT scan of the lung region from the patient.

To our knowledge, previous studies have mainly used traditional convolutional neural networks (CNNs) for image-based SRP. One major issue of these types of neural networks is that their extracted features strongly depend on the spatial orientation of the tumor. That is, a rotated tumor can potentially get a different prediction by using traditional CNN. Instead, spherical CNNs (SphCNNs) are designed to be invariant against changes in orientation. Thus, SphCNNs are theoretically better suited for SRP. While traditional CNNs work with inputs structured in well-defined Cartesian grids, SphCNNs work with functions defined on the unit sphere. Thus, the use of SphCNNs for SRP requires a mapping from 3D CT images to functions on the unit sphere, which are intrinsically 2D. This dimensionality reduction has the additional effect that the derived DL models are less prone to overfitting in complex tasks with small datasets of 3D images (24). These reasons make it interesting to assess the ability of SphCNNs for SRP.

The aim of this study is to propose a fully-automatic solution for SRP of cancer patient data. First, we train a deep learning-based model that is able to segment tumors from CT images automatically. In a second step, we use spherical convolutional neural networks (SphCNNs) to perform deep feature extraction for SRP. To our knowledge, such spherical features SphCNNs have not been used in this context before. Thus, we also compare our SphCNN-based pipeline against more traditional methods using different prediction models for SRP on radiomic features or features extracted from fine-tuned DL-based pre-trained classifiers.

The remainder of this paper is structured in the following way. Section 2 establishes a general framework for SRP consisting of three stages: tumor segmentation, feature extraction, and survival prediction. Next, we describe how our proposed pipeline implements each of those stages. Moreover, the implemented baseline methods are described. Section 3 lists the experimental results comparing the proposed method with the baseline models. Section 4 discusses the findings from the experimental evaluations. Finally, section 5 reveals the main implications of the results and makes some conclusions of the study.

## Materials and Methods

**Figure 1:**
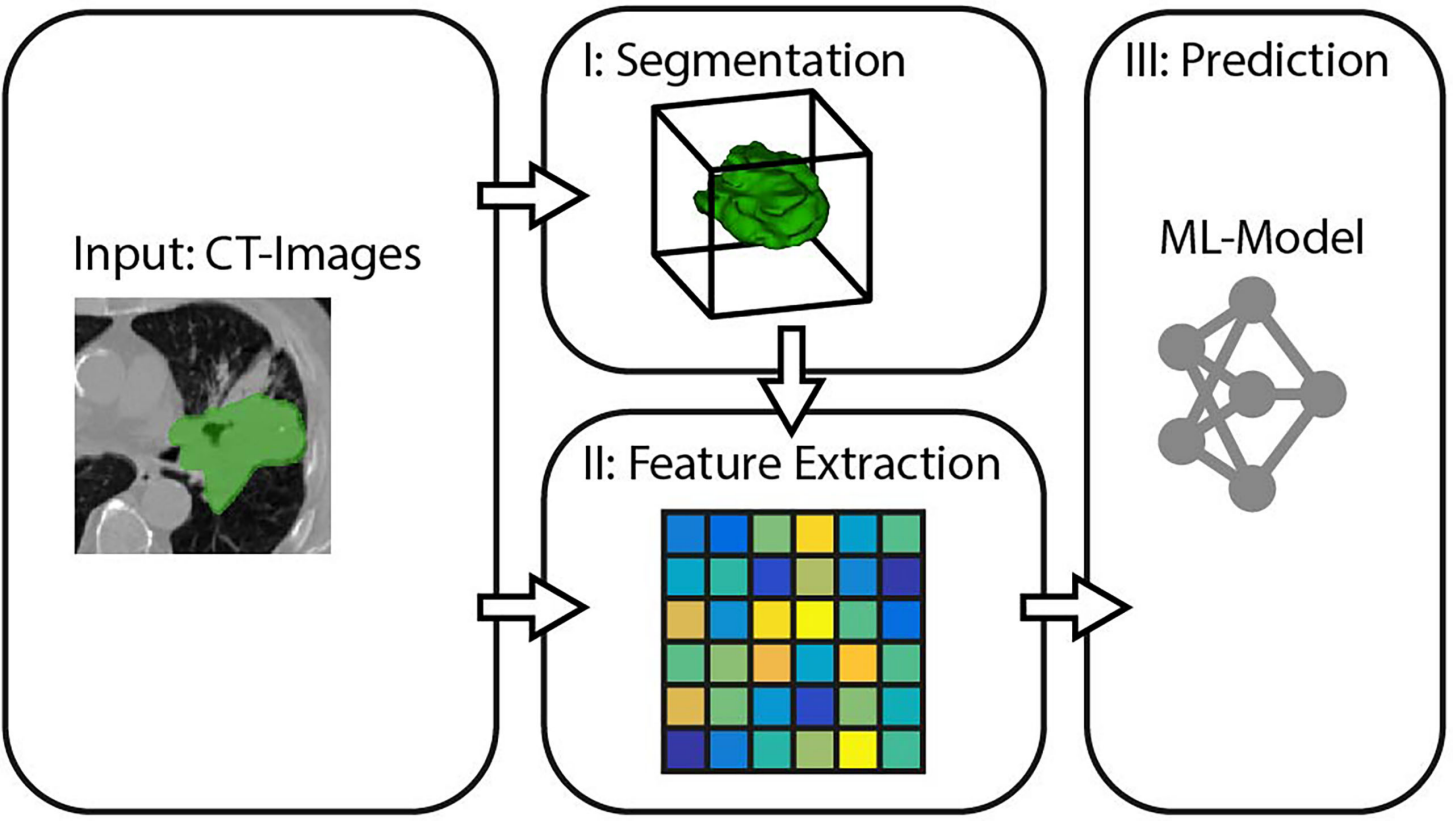
General pipeline for survival rate prediction.

In the context of this study, we model the SRP of cancer patients as a three-stage process, consisting of *segmentation*, *feature extraction*, and *survival prediction* (cf. **Figure 1**).

- **Tumor Segmentation** describes the process of defining which of the voxels belong to the object of interest, that is, which parts of the CT image depict the cancerous mass. Therefore, a binary mask is generated either by manual annotation through a medical expert or an algorithmic segmentation method.
- **Feature Extraction** is the transformation of high dimensional input data (in our case, segmented regions of the image) into fewer but more relevant features.
- **Survival Prediction** takes the previously extracted features and determines the respective value of interest.

We will refer to these three stages when comparing different prediction pipelines in the experiments. The following subsections specify the methods we propose for each of the three SRP stages.

### Tumor Segmentation

**Figure 2:**
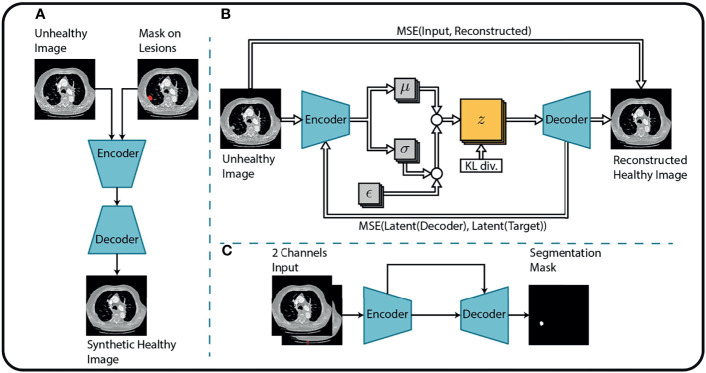
Overview of the tumor segmentation method. The segmentation method incorporates **(A)** an image inpainting network, **(B)** a variational autoencoder, and **(C)** a U-Net for the final segmentation. Replicated from (25) with permission from Springer Nature Switzerland AG.

This study aims to introduce an end-to-end solution for SRP that does not rely on manual tumor segmentations. Therefore, we incorporate a fully automatic lung nodule segmentation model, concretely, the lung cancer detection and segmentation method we proposed in (25). This method decomposes the segmentation problem into three separate steps, as shown in **Figure 2**.

First, an in-painting network (26) is trained to fill randomly generated holes in Lung-CT images from healthy subjects. The resulting network can fill missing parts of an image with semantically meaningful patterns. By considering the annotated tumor regions of the unhealthy images as missing content, the in-painting network is used to generate healthy synthetic images from the unhealthy counterparts.

Second, the resulting healthy-unhealthy image pairs are used to train a normal appearance autoencoder (NAA). Here, the unhealthy images serve as an input and the healthy synthetic images as corresponding target images for the supervised training of the NAA model. Therefore, the trained NAA can generate tumor-free images from arbitrary unhealthy images without depending on manual annotation masks.

In the final stage, the original (unhealthy) image and the difference between the original image and the NAA-generated healthy outputs are fed to a standard U-Net segmentation model. The U-Net model benefits from this attention cue to learn the final segmentation mask by receiving the original and difference-image as separate channels. The method is described in more detail in (25). Performance metrics of this method for the datasets that are relevant for this study are presented in **Table 1**.

**Table 1:** Performance metrics (mean ± std) of the automatic segmentation methods evaluated on two lung cancer datasets.

Dataset	Dice	Precision	Recall	Specificity
Lung1	0.77 ± 0.17	0.76 ± 0.20	0.82 ± 0.15	1.00 ± 0.00
Lung3	0.76 ± 0.18	0.74 ± 0.22	0.85 ± 0.16	1.00 ± 0.00

For the training dataset Lung1, the observed values are averaged over five evaluation folds. For the validation dataset Lung3, the values are averaged over all samples. Note that we rounded to two digits so 1.00 in the last column results from rounding a value close to one.

### Feature Extraction

This section discusses how our pipeline extracts descriptive variables that are meaningful for the prediction task from the raw data, i.e., the lung-CT images.

**Figure 3:**
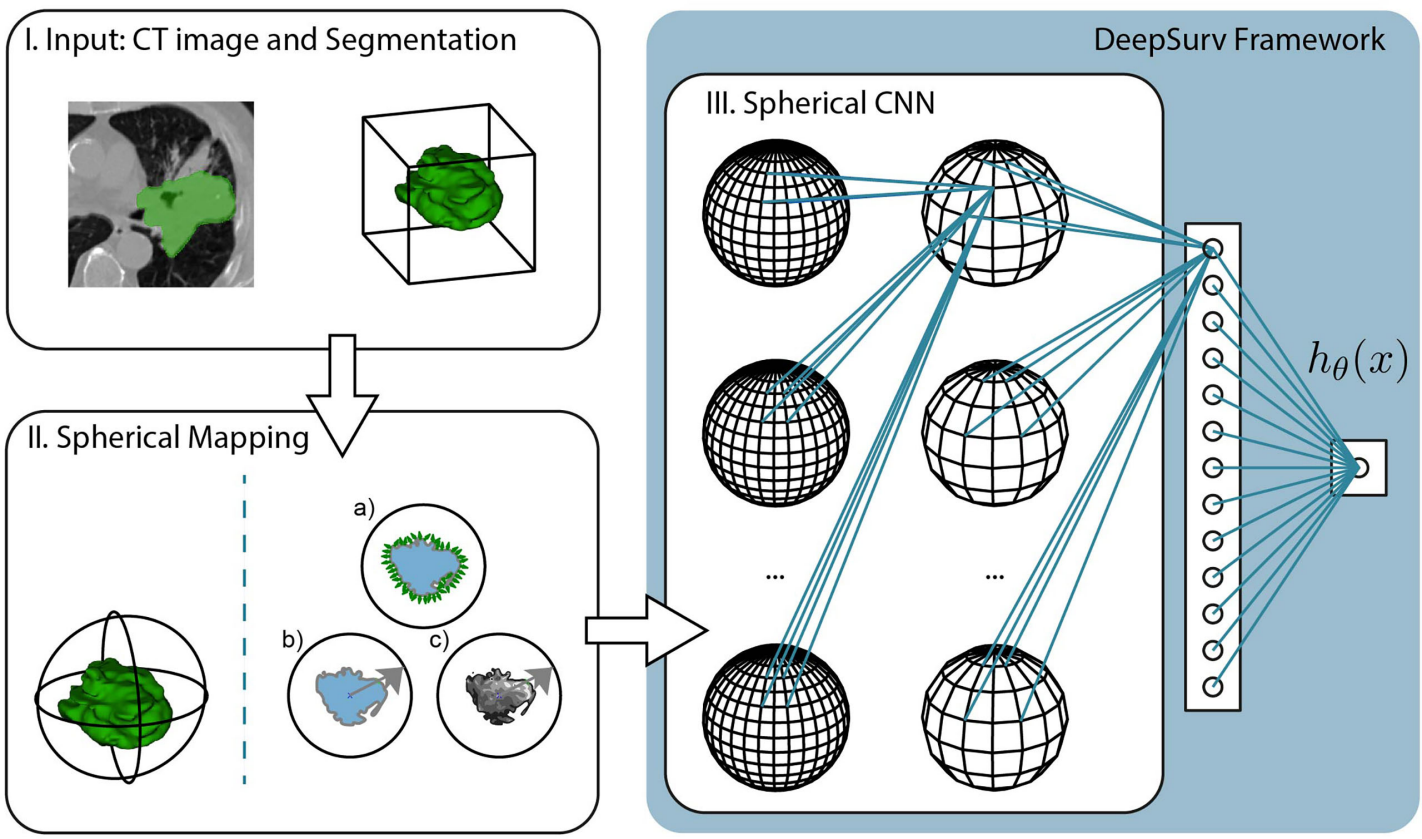
Pipeline of the proposed method, which is divided into three steps. I. The input consists of the CT image and segmentation of the tumor mass. The experiments compare the predictive performance of provided manual segmentation masks with our automatic segmentation. II. The volumetric images are projected into the spherical domain to be usable with Spherical CNNs. In this study, we propose three spherical mappings; a) the extended Gaussian image (EGI), b) the depth-based projection of the mask (b), and c) the spherical intensity mapping of the masked image content. III. The Spherical CNN consists of a cascade of spherical kernel stacks followed by spherical pooling operations. The Spherical CNN is embedded in the DeepSurv framework that includes a fully connected layer that pass the activation signal to a single output node. This scalar output is the approximation of the log-risk function *h_θ_* (*x*) in Cox proportional hazards model which is optimized through DeepSurv.

SphCNNs (27, 28) extend the standard operations used by traditional Cartesian CNNs to work on signals defined on the sphere. The network topology of SphCNN consists of stacks of spherical filters that are applied on the spherical activation signals *via* spherical convolution (cf. **Figure 3**). The convolution operation is often carried out as a multiplication in the spherical harmonics domain. One characteristic property of SphCNN is that it can be used for solving problems where rotational equivariance (i.e., the output rotates when the input is rotated) or rotational invariance (i.e., the output is always the same even if the input is rotated) is required (28). As mentioned, SRP should be rotational invariant, which means that the prediction should be the same regardless of the orientation of the tumor in the lungs. Our implementation builds upon the code provided in (27).

**Figure 4:**
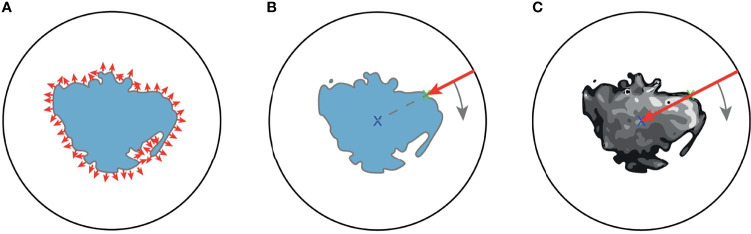
Illustrative depiction of the three proposed spherical mappings. Note that volumes are here drawn as image slices, and therefore, the spheres are depicted as circles. **(A)** The extended Gaussian image (EGI) can be viewed as an accumulation of the gradient vectors (small red arrows) at the surface of the tumoral boundary. **(B)** Depth-based projection of the solid segmentation mask. A ray (red arrow) is cast from a projecting sphere to the surface of the segmentation. The distance from the sphere to the surface determines the value of the spherical signal at the respective position. **(C)** Intensity-based projection of the voxel image content. A ray (red arrow) is cast from the surrounding sphere through the segmented tumor image towards the centroid. The value of the spherical signal is the sum of all intensities of the voxels that the ray traversed.

In order to apply SphCNN on volumetric CT images, it is necessary to map the segmented tumor onto the unit sphere *S* ^2^. We propose three different mapping methods for this projection, a) the extended Gaussian image (EGI) of the tumor mask, which is the orientation distribution function of the normal vectors from the surface of the tumor (29), b) a depth-based projection (30) of the segmentation mask, and c) an intensity-based projection of the tumor. As for the EGI, it is generated from the normal vectors derived from the provided manual segmentations or the generated automatic segmentation masks (cf. **Figure 4A**). Regarding the depth-based projection, first, an enclosing sphere is centered at the tumor’s center of mass. Next, a ray is cast from each sampling point on the surface of the sphere to the centroid. The distance to the first intersection point then decides the value of the spherical signal at that specific orientation (cf. **Figure 4B**). For an alternative mapping, we accumulate the intensity values within the tumor along every ray (cf. **Figure 4C**). These three functions on the sphere are used as input channels for the SphCNN. These three functions on the sphere are used as input channels for the SphCNN. Notably, we explore two configurations here; the first input configuration only uses the segmentations’ depth-based projection (later referred to as SphCNN[1]). The second uses the EGI and the intensity-based projection from the image (SphCNN[2] in the following). This choice of input channels is motivated by the question of whether the image content carries additional predictive power to the use of the segmentation mask alone.

Prior to the comparative experimental evaluation presented in the results section, we empirically determined a suitable network topology for our purpose. Those tests uncovered that a deeper SphCNN was not beneficial over a more shallow architecture for the given problem. Therefore, the best-performing model consists of three layers. The first convolutional layer lifts the input signal from the sphere, *S* ^2^ onto the SO (3) manifold. Next, the spherical activation maps are fed to another convolutional layer [operating on SO (3)] and, finally, a dense layer that connects *via* linear activation function to the scalar output neuron. Interposed spherical pooling layers condense the spatial dimension of the activation maps. The last fully-connected layer encodes 40 features. The configuration of the empirically determined training parameters used in the experiments is provided in **APPENDIX A**.

### Survival Prediction

In this paper, we aim to predict the relative risk of a patient and the chance of survival for different times. Every longitudinal entry in the clinical datasets records the observation time T and a binary event variable E, which indicates whether the event of death occurred at time T. T represents the actual survival time when E is equal to one (representing the state ‘True’). However, if E is equal to zero (representing the state ‘False’), the data entry is considered as *right-censored*, and T can only be seen as a lower bound for the actual unknown time of survival.

One possible approach to handle this type of data could be to disregard all data points with *E*≠1 and perform regression on the remaining data. However, as mentioned previously, this approach would bias the method towards the subjects with higher mortality. Instead, the problem of SRP under the presence of right-censored data is commonly modeled *via survivor-* and *hazard functions*.

The standard method of handling SRP on right-censored data is the *Cox’s Proportional Hazard* model (CoxPH) (31). Cox (31) defined the survivor function as *F*(*t*)=*P*(*T* ≥*t*), that is, the probability *P* of the actual death of the patient to be larger or equal to the time t. Cox also defined the hazard function *λ*(t) which models the age-specific failure rate as:


(1) $$\lambda \left(t\right)=\underset{\text{Δ}t\to 0_{+}}{\lim}\frac{1}{\Delta t}P\left(t\langle T\langle t+\text{Δ}t|t\leq T\right).$$

He proposed the *Cox proportional hazards model* (CoxPH) to approximate the hazard function as:


(2) $$\lambda (t|x)=\lambda_{0}\left(t\right)\cdot e^{h\left(x\right)}=\lambda_{0}\left(t\right)\cdot e^{\beta^{T}x},$$

Where *λ*_0_(t) is the (unknown) baseline hazard, β is the model parameter vector, *h*(*x*) is the so-called *log-risk* function, and *x* are covariates. Note that in our specific problem, the covariates are the features extracted from the sample, as discussed in the previous subsection.

One well-known restriction of the CoxPH is the assumption that *h*(*x*) is linear, i.e. *h*(*x*) = *β^T^x*, which can limit the capability of the function to model SRP. DeepSurv (11) tackled this problem by training a neural network to approximate *h*(*x*) which is able to model non-linearities in the hazard function. Thus, the hazard function in DeepSurv becomes:


(3) $$\lambda (t|x)=\lambda_{0}\left(t\right)\cdot e^{h_{\theta }\left(x\right)},$$

where *h* ≈ *h_θ_* (*x*) with *θ* being the learned parameters of the neural network.

One advantage of DeepSurv is that it is more than a method, it is a generic pipeline that can easily be connected to a feature extraction neural network. In the original paper, DeepSurv used a set of fully-connected layers followed by a linear combination layer to estimate *h_θ_* (*x*). Instead of fully-connected layers, we used the SphCNN described in the previous section while keeping the same loss function that aims to minimize the *average negative log partial likelihood* of *h*(*x*), as described in (11).

Beyond DeepSurv, a family of techniques that aim to address the shortcomings of CoxPH are large-margin methods such as regression or ranking-based support vector machines (SVMs) (32, 33). Other techniques that have been applied successfully for SRP are ensemble models that use, e.g., gradient-boosting to learn a partial likelihood function (34). Notice that these methods can only be used when the feature extraction is independent of the survival prediction model, which is not our case. Thus, DeepSurv is a well-suited choice for combining feature extraction and prediction simultaneously and is therefore used in the proposed method.

### Baseline Methods

In order to assess the relative performance of the proposed method, we compared it against multiple feature sets and prediction method combinations. As for the features, we computed radiomics features (RF) (5, 22) and deep learning-based 2D (slice-based) image features (DIF) (22). Instead of the pre-trained neural network used in (22), we used ResNet50 (35), which is very well-known for its good performance in transfer learning tasks. In particular, the DIF features were extracted from the 2D axial slice with the largest tumor area in the segmentation mask with the pre-trained ResNet50. The RF and DIF sets consist of ca. 1,500 and 1,000 features, respectively. Moreover, subsets of 32 features were extracted from RF and DIF after a feature selection procedure, which are referred to as RF32 and DIF32, respectively (more details are provided in **APPENDIX B**). For this, we used the library function from *scikit-learn* (36) to rank each regressor (i.e., each entry of the extracted feature vector) based on its cross-correlation with the target.

As survival prediction methods we used support vector machines with ranking (SVM-K) and regression (SVM-R) objective (32), CoxPH (31, 37), and the gradient boosting-based ensemble (EGB) model proposed in (34). Thus, we implemented the sixteen combinations of four features sets and four survival prediction methods. In addition, we implemented the method proposed by Aerts et al. (2) in which CoxPH is applied to the so-called radiomics signature that consists of four radiomic features. This method is referred to as RS-CoxPH in the experiments. Hyperparameters such as the learning rate or method-specific engineering values were empirically tuned in a set of preceding tests.

## Results

**Figure 5:**
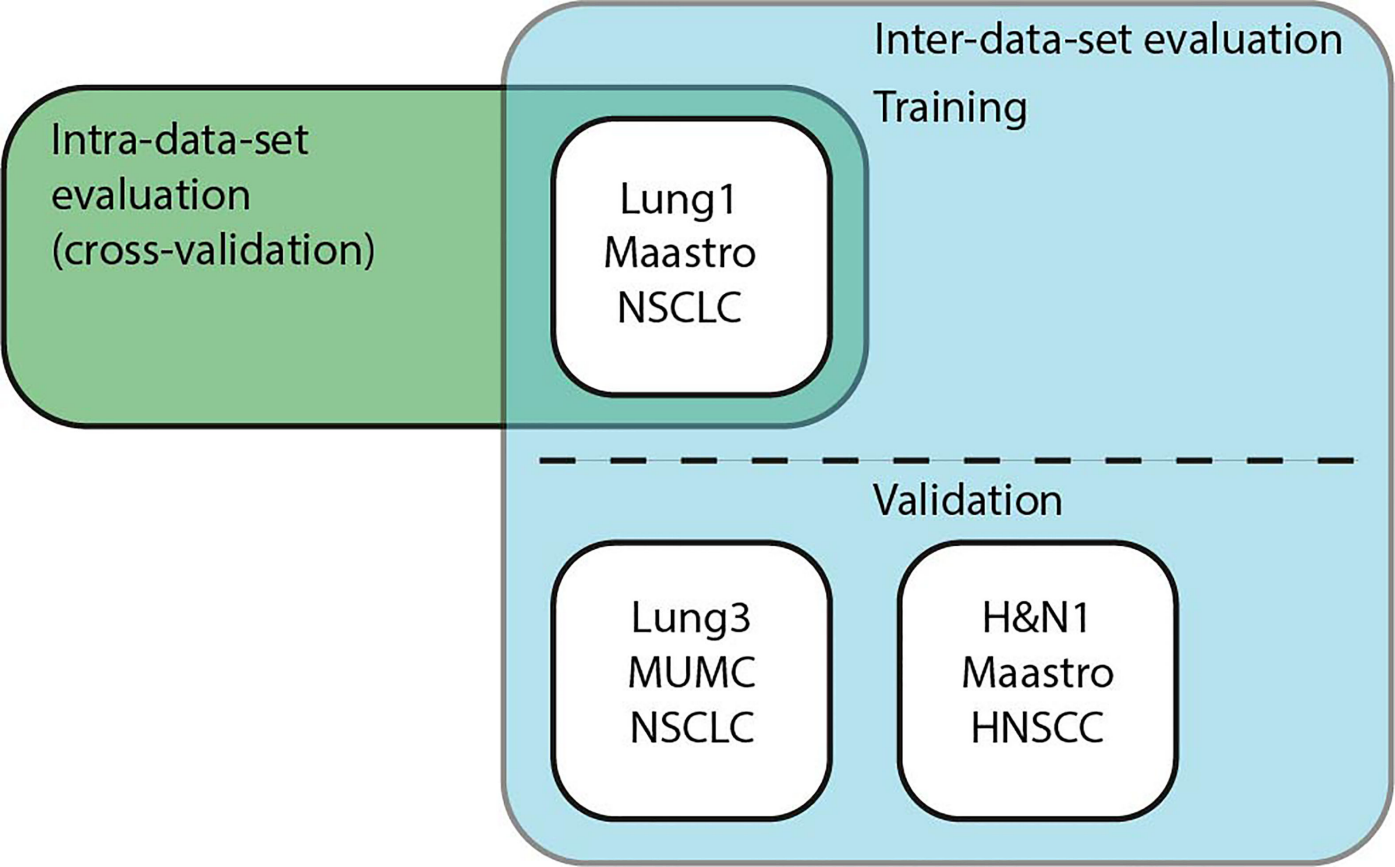
Schematic overview of the experiments. **Intra-data-set** uses Lung1 for training and testing in a cross-validation setup. For **Inter-dataset** evaluation, methods were trained on Lung1 and evaluated on Lung3 or H&N1.

We performed intra- and inter-dataset experiments with different pipelines and dataset configurations to assess the model performance and robustness of the different methods as shown in **Figure 5**. In particular, we trained our models on the CT data from the publicly available Lung1 (n=422) (38), Lung3 (n=89) (39), and H&N1 (n=136) (40) datasets. While Lung1 and Lung3 contains data from Non-Small Cell Lung Cancer (NSCLC) patients acquired in different institutions, H&N1 depicts head and neck cancer. We used as ground truth prediction values the right-censored times of survival that were reported from the respective data providers.

We used the concordance index (C-Index) (41) as our main performance criterion, which is commonly used for problems with right-censored data like SRP. The C-Index measures how good the survival times of a set of patients are ranked and can be seen as a generalization of the area under the receiver operating characteristic (ROC) curve (AUROC) that can take into account right-censored data. We used the implementation of the C-Index from the python library *scikit-survival* ^1^.

### Results for Lung1

Intra-dataset performance was assessed using 5-fold cross-validation on Lung1. We kept the fold splits consistent for all evaluated methods. In addition to the 17 baseline methods described in Subsection 2.4, we tested the proposed method with both manually annotated segmentation masks and automatic masks generated by the method described in Sect. 2.1.

**Figure 6:**
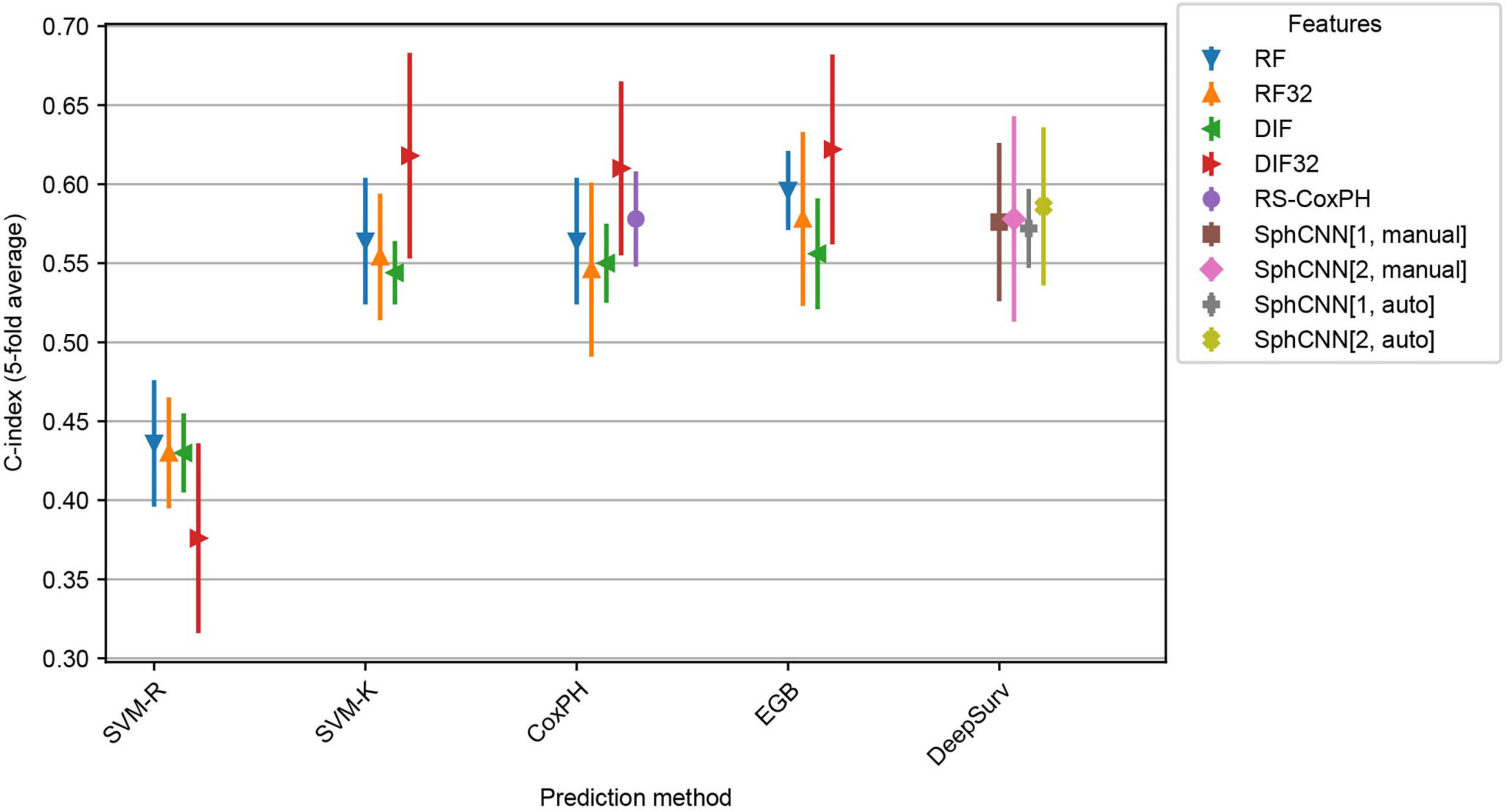
Cross evaluation results for 17 baseline SRP methods and the proposed one. The models were trained on four splits from the Lung1 Data and evaluated on the remaining fifth split. We report the average across the splits (marker) as well as the observed minimum and maximum observed values (line). Compared prediction methods are Support Vector Machines with regression (SVM-R) and ranging (SVM-K) objective, Cox Proportional Hazards model (CoxPH), Ensemble Gradient Boosting (EGB) and DeepSurv, a deep-learning-based prediction framework. Baseline features are Radiomics Features (RF) and pre-trained deep 2D Image Features (DIF). Both feature sets were also used with feature selection (RF32 and DIF32 respectively). In addition, we also include the Radiomics Signature (RS-CoxPH) suggested by Aerts et al. (2) in out comparison. Our proposed method uses a Spherical Convolutional Neural Network (SphCNN) with manual (SphCNN[…, manual]) and automatic (SphCNN[…, auto]) tumor segmentation. The spherical input for the SphCNN is either extracted *via* depth-image projection from the segmentaion mask (SphCNN[1,…]) or composed of intensity projection and extended gaussian image from the CT-image (SphCNN[2,…]).

**Figure 6** shows the observed C-indices of the 5-fold cross-validation experiment. As shown, the combination of EGB and DIF32 obtained the best performance with a C-index of 0.62 ± 0.04 in this experiment, while the worst performance was measured on SVM-R with DLF32: 0.38 ± 0.04. In comparison, the proposed method achieved 0.58 ± 0.04 for the manual masks and 0.59 ± 0.03 for the automatic ones.

### Inter-Dataset Evaluation

In order to assess the robustness of the methods, inter-dataset validation was carried out by training the methods (including the automatic segmentation) on Lung1 and validating on additional images from a different dataset. In particular, we used the models that were fitted to Lung1 for inference on two independent datasets: Lung3 and H&N1. As mentioned, Lung3 has the same type of patients (i.e. NSCLC-patients), while H&N1 contains images of patients with head and neck cancer.

**Figure 7:**
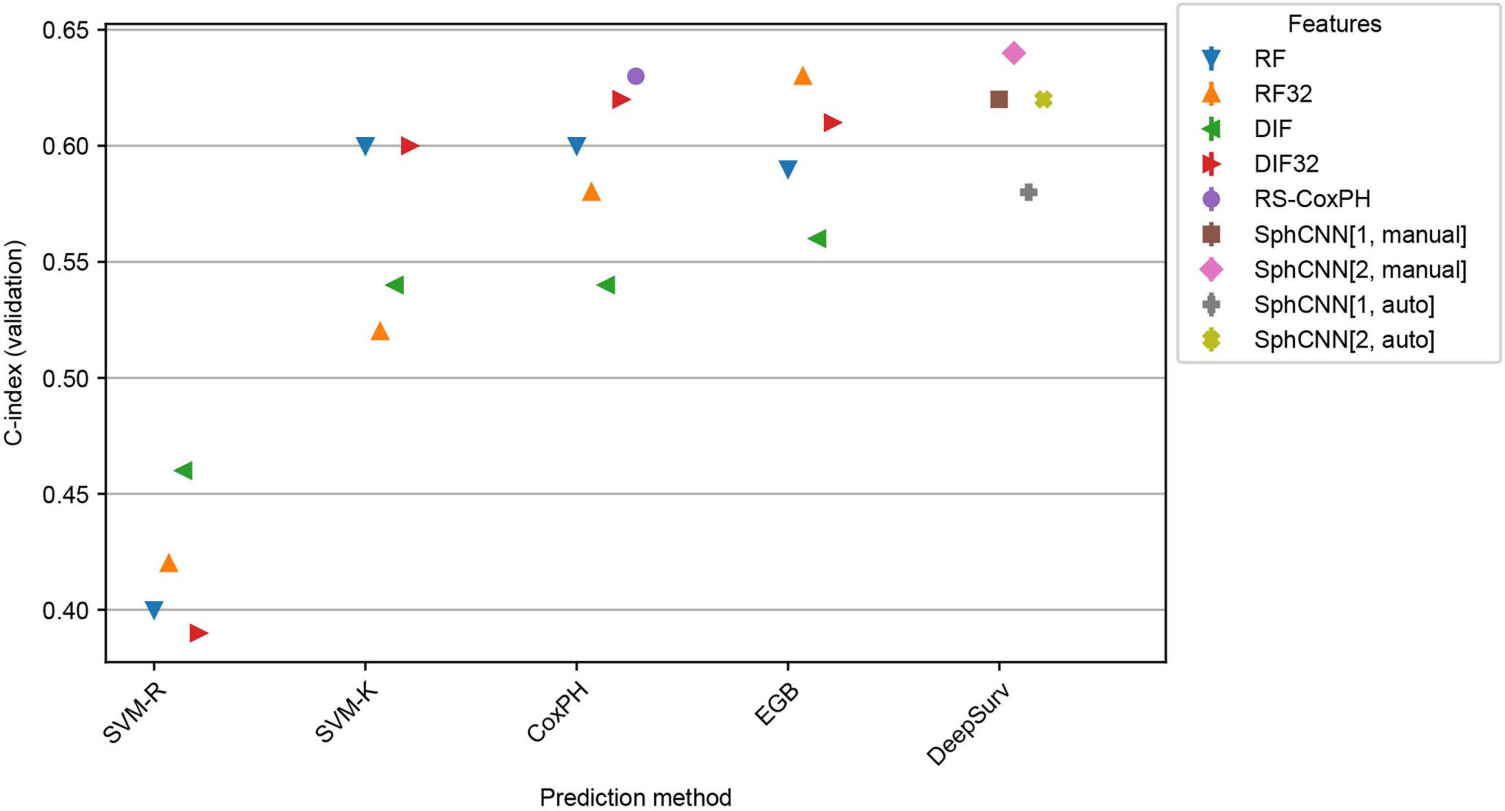
Performance comparison for the models trained on Lung1 and evaluated on Lung3. We used the prediction methods and features as labelled in **Figure 6**.

**Figure 8:**
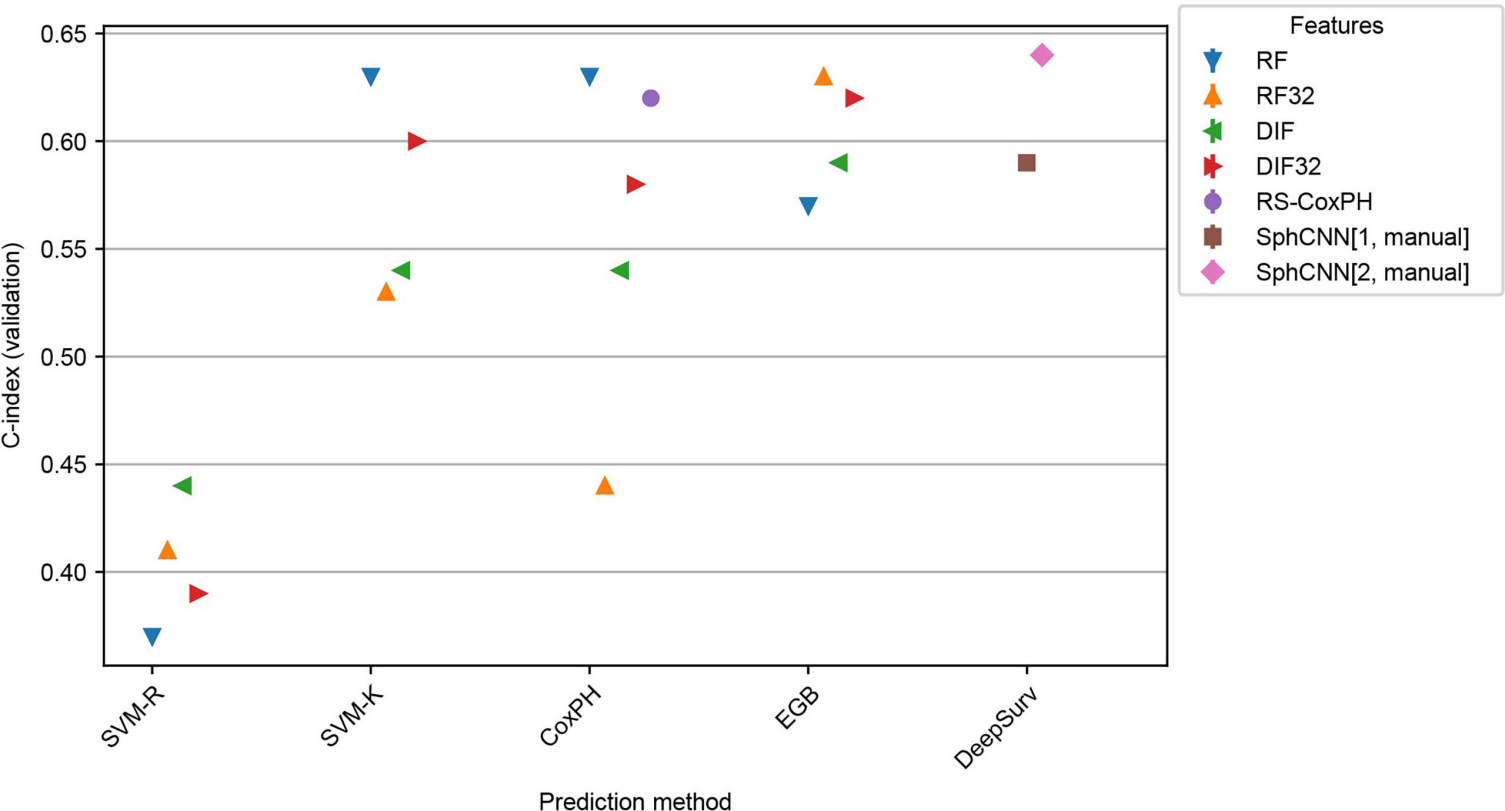
Performance comparison for the models trained on Lung1 and evaluated on H&N1. We used the prediction methods and features as labelled in **Figure 6** with the exception that no automatic segmentation was evaluated here.

Results of these experiments are reported in **Figures 7** and **8**. The best-performing method was the proposed one (C-index 0.64 both for Lung3 and H&N1), followed by EGB with RF32 (C-index 0.63 both for Lung3 and H&N1). EGB with DLF32 - the best method in the previous experiment - decreased its performance in this test to 0.61 for Lung3 and 0.62 for H&N1, respectively.

Since our automatic segmentation method was trained and developed for lung cancer, it did not yield meaningful segmentation results for CT images from head and neck regions. Actually, it is well-known that automatic segmentation of head and neck cancer is a very difficult task (42). Thus, our methods were tested only with the manually annotated masks provided in the datasets.

As shown in the experiments, the proposed method performed better when all spherical mappings were used. As expected, the proposed method yields slightly better results with manual segmentations compared to the use of automatic segmentation.

### Kaplan-Meier Analysis

**Figure 9:**
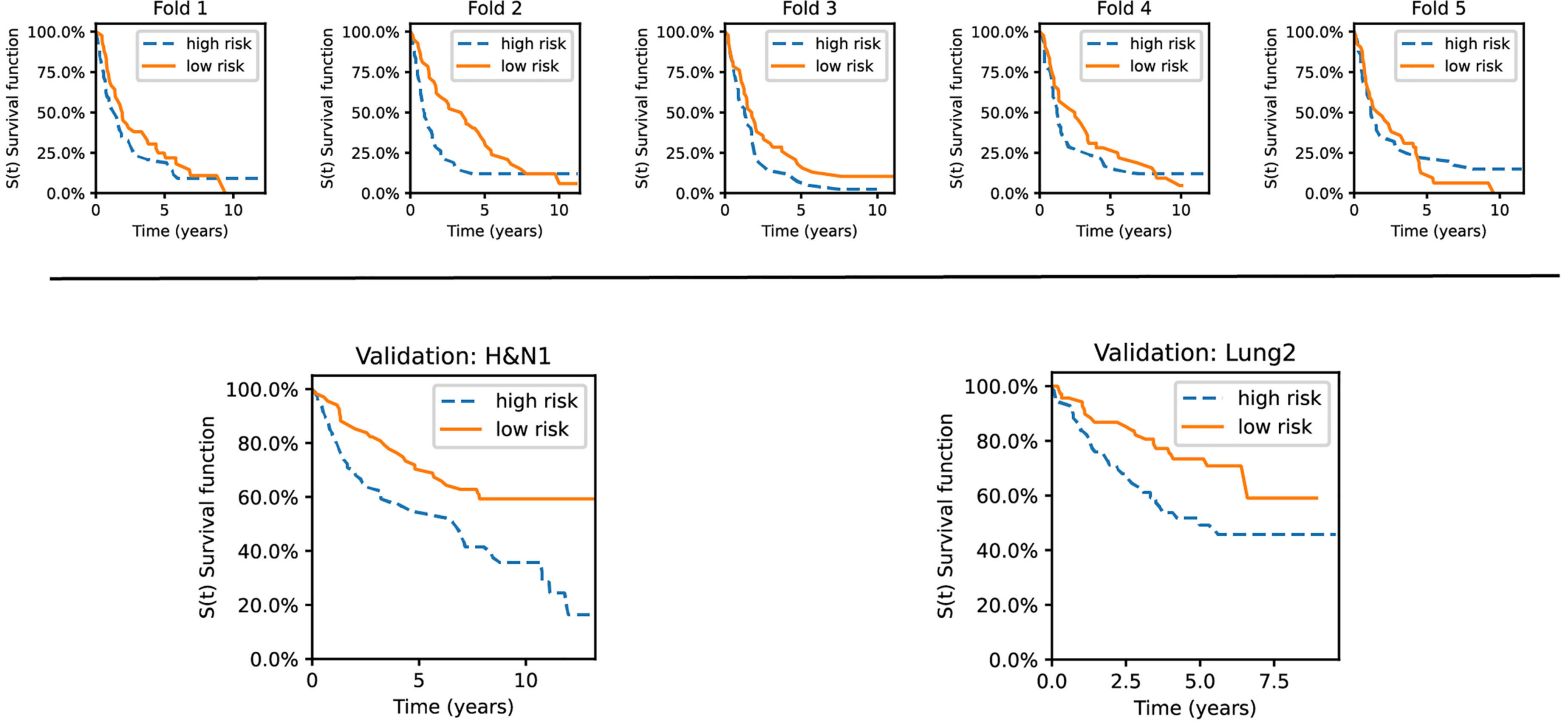
Kaplan–Meier curves for the validation sets used in the experiments. The datasets are stratified into low- and high-risk groups based on the risk predictions of our best-performing method. Top row: survival curves from five individual folds from Lung1. Bottom: The model was trained on Lung1 and evaluated on the Lung3 (right) and H&N1 (left) datasets, respectively.

Validation datasets from the respective experiment were stratified according to our best-performing method’s assigned risk score (i.e., SphCNN[2]). The stratification into risk groups was done based on the median of the predicted risk scores. Therefore, half of the higher-risk samples were binned into one group and the other half into another. Then, non-parametric Kaplan-Meier (KM) estimations were evaluated on each group separately (cf. **Figure 9**).

As shown, the KM curves also reflect the relative performance assessment reported in the previous subsections. Concretely, the evaluation folds of the cross-validation experiments reported mixed observations regarding their performance. In contrast, our method showed promising stratification abilities when tested on the external datasets (Lung3, H&N1). There is also a potential trend observable regarding the type of cancer. Lung cancer data has good short-term separability but often fails for long-term prediction (over five years). In contrast, head and neck cancer data showed more confident separation (i.e., the survival curves become more distant from each other) for periods larger than ten years.

## Discussion

The primary goal of this study was to introduce a novel fully-automatic lung cancer SRP model based on CT-images. In addition, we performed a benchmark with various SRP models evaluated on publicly available data. We run our experiments in both inter and intra dataset evaluation schemes. This section discusses some findings and analytical aspects of the presented investigations.

Perhaps the most apparent observation is that the C-index values from SVM-R scored lower than all other methods across different features. This difference in prediction performance confirms the previous claim that regression methods are poorly fit for working with right-censored data. Historically, this misfit motivated the development of ranking and hazard-based SRP models. Thus, we will focus the discussion on the remaining prediction methods.

In contrast, we found that pre-trained ResNet50 with feature selection (DIF32) had the highest predictive power in the cross-validation experiment. Notice that the original set of features (DIF) did not perform well in the experiments. That means that the boost in performance is mainly due to the feature selection method. We like to emphasize that the reported use of DIF with feature selection entails the risk of overfitting the given training set. Since only the 32 highest-rated regressors were selected on the training data, the possibility arises that this selection might be biased towards the respective training dataset. By using additional validation datasets, we confirmed the predictive power of this model but, in that case, we did not observe any advantage of this method over the other tested methods. In comparison, the proposed method used 40 features that, according to the results, are enough to encode the most relevant information for SRP. Thus, the results support that a small set of spherical features or DIF are beneficial for SRP.

Regarding radiomic features, feature selection resulted in worse performance except for EGB in the inter-dataset evaluation. In specific, it should be noticed that the employed cross-correlation based feature selection method aim to hold only those subset of the features that are more linearly correlated with respect to the class labels statistically. However, this linear statistical association does not necessarily represent their more prognostic values of the feature subsets. Accordingly, although the selected subset of radiomic features is more correlated with the target values, their prediction power is not as high as the whole radiomic feature set. In addition, the observation that such a feature selection method leads to improving the performance of DL-based features but not the radiomic features can be explained by the fact that DL-based features were extracted from a single 2D slice, i.e., the central tumoral slice, while the radiomics descriptors were extracted from the tumor volumes. Therefore, the less complicated attributes of the tumors in 2D slices which were captured by the DL model, are more prone to show stronger association with the target labels compared against the 3D radiomics descriptors that were extracted from the irregular tumor volumes with a large variety of texture, intensity, and morphological characteristics.

Furthermore, we noted that except for SVM-R, all evaluated prediction methods resulted in C-indices at a comparable level. In our experimental setup, the individual remaining prediction methods do not seem to have an advantage over other prediction methods. Besides, the differences in the feature extraction methods were mostly consistent for different prediction models and datasets. Therefore, we observe that the choice of extracted features is much more important for designing a successful SRP pipeline than the selection of the prediction model, as long as they are designed to handle right-censored data.

Our proposed method automatically extracts morphological features in the spherical domain. Intuitively, our spherical mapping methods can be understood as a compact representation of tumor surface texture, size, shape, and internal structure. Using such spherical signals combined with a rotation-invariant SphCNNs, we obtained C-indices comparable to conventional methods on the cross-validation experiment. Moreover, the proposed method slightly surpassed the others when referring to the inter-dataset evaluations. Our results suggest that the proposed SphCNN-based SRP is robust when applied to new, unknown datasets. The observed statistics also indicate a similar accuracy on both the lung cancer data and the head and neck images. This finding hints that the morphological features that the SphCNN internalized during training might have prognostic relevance for tumors in general. However, since the differences between the proposed method and the best-performing baselines were small, we can only argue that the proposed method has overall competitive performance.

In clinical settings, it may be difficult or unfeasible to have high-quality annotated segmentation masks of the tumors. For that reason, it is relevant to have a fully automated solution that includes an automatic segmentation tool. Since manual annotations performed by experts have higher quality than segmentations from automatic tools, we expected a reduction in the performance of the proposed SRP method when used on automatically segmented tumors. From the results, such a reduction was slightly negative for the intra-dataset experiment (0.58 ± 0.04 vs. 0.59 ± 0.03) and very small for the inter-dataset evaluation (0.64 vs. 0.62). This means that our proposed pipeline can yield similar results when it is run autonomously without a manual - and potentially expensive - human intervention.

To gain insights if the segmentation mask or the segmented image channels are beneficial for SRP, we tested our method in two different configurations, SphCNN[1] and SphCNN[2]. While the former represents a higher compressed version of the signal, the latter is assumed to preserve more structural information. Our reported results support the assumption that SphCNN[2] is slightly superior in this context.

As mentioned, DeepSurv is a general pipeline that can potentially be combined with any feature set, including RF and DIF. We did not include these combinations in the experiments since that would require a fine-tuning of the architecture of the neural network for every specific feature set, which is out of the scope of this study.

Since the implementation of methods by different research groups can yield different results, we decided to implement 17 baseline methods in order to have a more fair comparison. The performance of all tested methods was below 0.65, which is consistent with previous studies [e.g (2, 21).,]. That means that, although CT images convey important information for SRP, they should be complemented with other types of information to improve the predictions to a level that can be used in clinics.

### Limitations of the Study

The main limitation of the study is the number of available images. It is well-known that DL-based methods require large datasets that are relatively scarce in cancer research at present. Thus, the main findings of this study require further validation with larger datasets. That could help to rule out the possibility that the differences in performance are related to the specific characteristics of the datasets. In this study, we avoided overfitting by using two strategies: a) dimensionality reduction by mapping the 3D data into 2D spherical mappings and b) the architecture of the proposed SphCNN is relatively small and has just 40 features in the penultimate activation. While dimensionality reduction will always be beneficial and needed, using larger datasets would enable us to evaluate larger SphCNN architectures with more parameters bigger feature vectors and overall higher capacity.

Another potential downside of the proposed solution is the representation of the spherical images and activation functions. The spherical signals are represented as a regular 2D grid in the implemented pipeline. While this common practice allows easy integration into the SphCNN framework, it might introduce distortions in the image due to the lack of equidistant sampling on the sphere. Concretely, regions close to the poles are oversampled compared to the equator. An alternative approach that samples the sphere more uniformly is described in (43). It is unclear at this point if and how this change of sampling can affect the predictions; therefore, more research would be required.

## Conclusion

This work introduced a new method for image-based lung cancer SRP. For automatic, relevant feature extraction, we mapped the tumor extracted with a DL-based method into a spherical domain and used SphCNN for prediction. The experimental evaluation confirmed the competitive predictive power of our model when compared to state-of-the-art approaches on the Lung1 data. A slight advantage over the other techniques was observed when tested on data from additional datasets (Lung3, H&N1). The results support that SphCNNs are helpful for attaining rotational invariance in SRP problems.

## Data Availability Statement

The original contributions presented in the study are included in the article/supplementary material. Further inquiries can be directed to the corresponding author.

## Author Contributions

FS: Conceptualization, Methodology, Implementation, Formal analysis, Writing - original draft, Visualizations. RM: Conceptualization, Methodology, Writing - review and editing, Supervision, Project administration, MA: Methodology, Implementation, Writing - review and editing, ÖS: Writing - review and editing, Supervision, Resources, Funding acquisition. All authors contributed to the article and approved the submitted version.

## Funding

This study has partially been funded by the Swedish Research Council, [grant no. VR-2018-04375] and the Swedish Childhood Cancer Foundation [grant no. MT2016-0016]. The funding agencies had no role in research or preparation of this study.

## Conflict of Interest

The authors declare that the research was conducted in the absence of any commercial or financial relationships that could be construed as a potential conflict of interest.

## Publisher’s Note

All claims expressed in this article are solely those of the authors and do not necessarily represent those of their affiliated organizations, or those of the publisher, the editors and the reviewers. Any product that may be evaluated in this article, or claim that may be made by its manufacturer, is not guaranteed or endorsed by the publisher.

## Footnotes

1
https://scikit-survival.readthedocs.io/en/stable/api/metrics.html

## APPENDIX A.Training Configuration of the Proposed Method

The training configuration of the SphCNNs used in this study is reported in **Table 2**.

**Table 2:** Configuration of the SphCNN prediction models.

Batch-size:	32
Number of epochs:	1000
Learning-rate:	Epoch 0-500: 1e-3 - 1e-5 (decreasing), Epoch 500-1000: 1e-5 (constant).
Optimizer:	ADAM
Dropout:	Yes, Rate 0.01
Batch-normalization:	Yes
Normalize inputs:	Yes

### APPENDIX B. Feature Selection for Baseline Methods

Motivated by the study of Aerts et al. (2), we decided to add a basic feature selection approach to our baseline feature extraction methods. The basic idea of feature selection in this context is to reduce the size of the feature vector that is presented to the prediction model for better convergence. Therefore, the cross-correlation of the features with the ground truth was measured on the respective training set. Next, only the features with the top 32 correlation scores were selected for prediction. The number 32 was selected to have a similar order of magnitude to the deep spherical features (i.e., the number of neurons in the penultimate layer of the SphCNN). Other feature vector sizes might lead to different results.

#### B.1. Feature Selection for Radiomics Features

In (2), features were selected based on their score within one of four categories (tumor intensity, tumor shape, tumor texture, and wavelet). In contrast, we score the cross-correlation as mentioned above on the complete set of approx 1500 radiomics features. A posthoc inspection of the selected radiomics revealed that the biggest group of selected features (18 out of 32) were first order-based statistics (mainly energy and total energy from different wavelet levels). The next biggest group were Gray Level Run Length Matrix components (8 out of 32), followed by Gray Level Size Zone Matrix entries (6 out of 32). For a detailed explanation of the different types of radiomics features, we refer to the documentation of *pyradiomics*.^^2^^

#### B.2. Feature Selection for Deep ResNet50 Features

Feature extraction *via* a pre-trained ResNet50 model transforms the CT-images slices into feature vectors of length 1000. The auxiliary hypothesis is that the ResNet50 model is equipped with 2D filter stacks that extract meaningful information for general image processing tasks. Since some of the tasks that the model was previously trained for might be unrelated to the prediction problem at hand, we test a possible reduction of the included features. For consistency with the radiomics baseline, the previously described feature selection method is used to reduce the size of the prediction input to 32. Unlike the features selected from radiomics, deep ResNet50 features are extracted by the pre-trained model and therefore do not carry interpretable labels.
